# Pressure Sensitivity of UiO-66 Framework with Encapsulated Spin Probe: A Molecular Dynamics Study

**DOI:** 10.3390/molecules30102247

**Published:** 2025-05-21

**Authors:** Dmitry V. Alimov, Artem S. Poryvaev, Matvey V. Fedin

**Affiliations:** 1 International Tomography Center SB RAS, 630090 Novosibirsk, Russia; d.alimov@tomo.nsc.ru (D.V.A.); poryvaev@tomo.nsc.ru (A.S.P.); 2 Physics Department, Novosibirsk State University, 630090 Novosibirsk, Russia

**Keywords:** molecular dynamics, spin probe, nitroxide radical, mechanical pressure sensor, metal–organic frameworks, UiO-66, EPR spectroscopy

## Abstract

Probes sensitive to mechanical stress are in high demand for analyzing pressure distributions in materials. Metal–organic frameworks (MOFs) are especially promising for designing pressure sensors due to their structural tunability. In this work, using classical molecular dynamics (MD) simulations, we clarified the mechanism of exceptional pressure sensitivity of the material based on the UiO-66 framework with a trace amount of spin probes encapsulated in cavities. The role of defects in the MOF structure has been revealed using a combination of electron paramagnetic resonance (EPR) spectroscopy and MD calculations, and potential degradation pathways under mechanical stress have been proposed. The combined MD and EPR study provides valuable insights for further development of new MOF-based sensors applicable for non-destructive pressure mapping in various materials.

## 1. Introduction

The molecular probes capable of detecting mechanical strain are actively developed in various fields of science and technology, especially in polymer science [1,2,3,4,5,6]. The most promising non-invasive methods for detecting mechanical stress by molecular probes include mechanochromism, chemiluminescence, and mechanofluorochromism [6,7,8,9].

However, despite their high potential, these approaches feature some challenges and limitations. For instance, they often require the incorporation of mechanophores as crosslinkers between polymer chains; in addition, they typically demand that the polymer matrix be optically transparent. The use of mechanophores as crosslinkers not only introduces additional synthetic steps but also alters the bonding characteristics within the mechanophores, which may prevent accurate representation of the mechanical breakdown processes in polymers [7,8].

An alternative and promising strategy involves the implementation of metal–organic framework (MOF) additives as mechanosensitive components. Mechanosensitive MOF particles can be dispersed in various polymers [10], potentially circumventing the constraints associated with traditional mechanophore-based systems. However, MOFs themselves represent only mechanosensitive particles and do not act as pressure probes, i.e., an analytical method is required to extract meaningful information about mechanical stress imposed on MOFs. Electron paramagnetic resonance (EPR) spectroscopy provides one of the most sensitive approaches for monitoring structural changes in MOFs and elucidating the local environment inside the pores of MOFs [11,12,13,14,15,16,17,18,19].

UiO-66 is one of the most broadly studied MOFs, whose applications in various fields of science have already been developed [20,21,22,23,24,25,26,27,28,29,30,31]. In our previous work, we discovered an unusual sensitivity to mechanical pressure demonstrated by a spin probe TEMPO encapsulated in the UiO-66 MOF (denoted below as TEMPO@UiO-66). The response of TEMPO@UiO-66 to pressure was monitored via EPR spectroscopy, and partial collapse of the MOF structure was observed already at mechanical pressure as low as 0.06 GPa, causing irreversible and significant changes in the EPR spectrum [32]. This observation was quite surprising because previous powder X-ray diffraction (PXRD) studies revealed only negligible changes in UiO-66 under the same pressure conditions [32,33,34]. At the same time, changes in the EPR spectra of the spin probe usually strongly correlate with the structural integrity of the MOF under various external stimuli, such as mechanical stress or chemical agents [35,36,37]. Therefore, the observed high pressure sensitivity of TEMPO@UiO-66 with EPR detection was unexpected but is a highly promising finding for designing new pressure-sensitive sensors.

The different pressure sensitivities of TEMPO@UiO-66 observed by EPR and PXRD, along with the lack of a clear explanation for such differences, make this system a subject of particular interest for further investigation. Spectral simulations suggest that, prior to pressure application, the nitroxide radical TEMPO undergoes characteristic fast mobility inside the pore, with a rotational correlation time being ca. 1 ns. After pressure was applied to a sample, two distinct fractions of the radical were observed: one in a fast-rotating regime and another one in a slow-rotating regime. Furthermore, even the fast-rotating fraction exhibited a noticeable slow-down of rotation after pressurization, showing a higher rotational correlation time of ~2–3 ns [32].

Understanding the spatial arrangements of the spin probes within the MOF cavities and identifying the factors responsible for their behavior is essential to elucidate the mechanisms behind their high pressure sensitivity. To gain comprehensive molecular-level insights into this process, the detailed molecular dynamics (MD) simulations of TEMPO@UiO-66 for various modifications of the ideal MOF structure are necessary. Therefore, in this work, we investigate the influence of various structural modifications of UiO-66 on the mobility of TEMPO radical in the pores of this MOF. Below, we demonstrate that the comparison of MD simulations with EPR data allows one to identify the most probable localization sites for the nitroxide and propose the mechanism of exceptional sensitivity of TEMPO@UiO-66 to mechanical stress.

## 2. Results and Discussion

**Figure 1:**
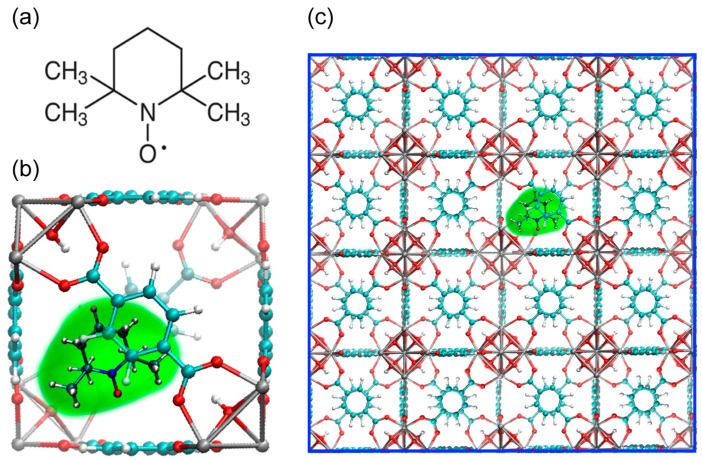
(**a**) Structure of nitroxide radical 2,2,6,6-tetramethylpiperydine-N-oxyl (TEMPO). (**b**) TEMPO encapsulated in UiO-66 cavity. (**c**) The model 4 × 4 × 4 cell structure of TEMPO@UIO-66 system investigated by MD simulations.

Figure 1 introduces the TEMPO@UiO-66 material under study, showing the structure of the nitroxide radical 2,2,6,6-tetramethylpiperydine-N-oxyl (TEMPO), sketching its encapsulation in UiO-66 cavity, and presenting the general 4 × 4 × 4 cell structure of TEMPO@UIO-66 that is used in MD investigations (vide infra). The cavity size of UiO-66 containing TEMPO is approximately 1.1 nm [38].

Previous studies have shown that pristine UiO-66 exhibits exceptional stability in its apparent MOF structure when subjected to mechanical stress up to 2 GPa [34]. However, the nitroxide radical TEMPO encapsulated in UiO-66 in trace amounts (1 radical per ~340 zirconium clusters, TEMPO@UiO-66) demonstrates a profound sensitivity to mechanical pressure (Figure S2) even at only 0.06 GPa [32]. It is known that the presence of defects within the MOF structure often leads to a decrease in the overall stability of the framework [34,39,40]. Therefore, the remarkable sensitivity of TEMPO@UiO-66 material to applied pressure can potentially be explained by the localization of spin probes in defective (and thus more fragile) cavities. To analyze the behavior of the radicals in these defective cavities by MD calculations, we introduced defects into the ideal model UiO-66 structure and assessed their impact on the mobility of the spin probe.

The most common type of defect in UiO-66 is the missing linker defect [41,42,43,44]. Previous studies of UiO-66 [34,41,42,43,44] and TEMPO@UiO-66 also indicate the presence of a certain number of missing linkers, whose amount varies depending on the synthesis procedure. For example, thermogravimetric analysis (TGA) of TEMPO@UiO-66 shows up to 1.65 missing linkers per Zr cluster on average (equivalent to 2.5 missing linkers per cavity), whereas the ideal UiO-66 structure contains 6 linkers per Zr cluster [34,41,42,43,44].

**Figure 2:**
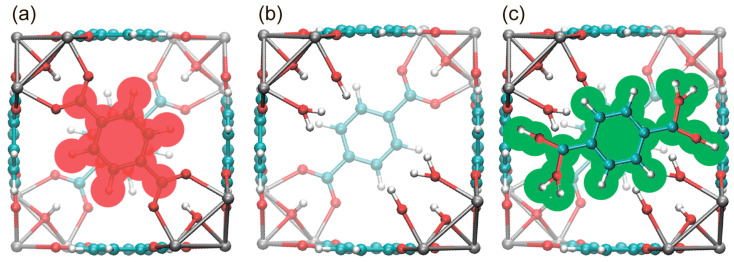
(**a**) Cavity of UiO-66. Removable linker is shaded in red. (**b**) Cavity of UiO-66 with a “missing linker” defect. (**c**) Cavity of UiO-66 with a “missing linker” and additional terephthalic acid molecule (shaded in green).

Therefore, we generated a series of model TEMPO@UiO-66 structures for subsequent MD calculations, in which different numbers of linkers were removed from the ideal UiO-66 cavities. To maintain the neutral charge of the entire MOF structure, we added -OH/H_2_O groups to the Zr atoms that have missing linkers. In addition to the structures with missing linkers, we also generated a set of structures where molecules of unbound terephthalic acid were added to the defective cavities. An addition of terephthalic acid molecules models the most typical decomposition product that might contaminate the cavity when the structure collapses, and it is based on the following considerations: (i) mechanical stress has inhomogeneous distribution in media [45], and (ii) the bonds between terephthalic acid and zirconium cluster are weaker than all other bonds in the MOF structure. Hence, mechanical stress might cause the onset of damaged regions/domains in the MOF structure, with the main destruction products being unbound (or partially unbound) terephthalic acid and zirconium oxocluster with different amounts of missing terephthalate linkers (from zero to three per zirconium oxocluster). If totally unbound, terephthalic acid is able to diffuse through UiO-66 pores and thus become the most likely final product of MOF structure degradation found in TEMPO-containing cavities (Figure 2).

Thus, in our modeling, two modifications of MOF cavities were considered as follows:

(I) Defective Cavities. In this case, an ideal cavity (Zr_6_(OH)_4_(BDC)_6_)_1_._5_ that contains six clusters (each belonging to the four cavities) was modified to introduce from one to three missing linkers. It is important to note that MOF structures with one or two missing linkers per cavity are the most common structural units obtained in as-synthesized UiO-66 [44]. At the same time, cavities with three missing linkers per cavity are considered strongly defective and may be formed under mechanical stress. A key feature of these defective cavities is the increased free volume, which facilitates TEMPO movement. However, at the same time, these cavities have extra active sites (OH/OH_2_ groups at Zr atoms) that enable interaction with the nitroxide radical and can potentially slow down its rotation.

(II) Contaminated Cavities. These cavities might be formed under mechanical stress and have no or some missing linkers, but, in addition, they are contaminated by terephthalic acid (TA) molecules. It is assumed that the contaminating TA molecule decreases the free volume available for nitroxide mobility and might, in addition, introduce some specific interactions. The number of introduced TA molecules was kept similar to the number of missing linkers in each cavity to model balance during the decomposition process. Altogether, this resulted in systems containing from one to three missing linkers, each compensated by an unbound TA molecule.

Using these two model structures of TEMPO@UiO-66 (I–II), we investigated the mobility and preferential sorption sites of TEMPO in these types of cavities via MD modeling. To explore the correlation between MD and EPR data, we analyzed the rotational correlation time for the nitroxide radical. The rotational correlation time (τ_c_) is a commonly extracted parameter from EPR spectra [46], and it undergoes significant changes when mechanical stress is applied to TEMPO@UiO-66 [32]. In MD simulations, the rotational correlation time was determined by fitting the decaying exponential of the autocorrelation function for the radical fragment’s vector, directed along the N-O bond.

**Figure 3:**
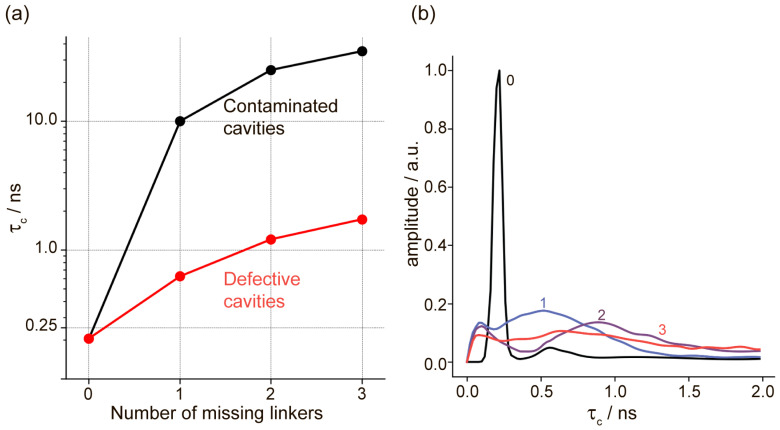
(**a**) Rotational correlation time (τ_c_) of TEMPO in defective UiO-66 cavities (red) and contaminated UiO-66 cavities (black). In both cases, the τ_c_ value is plotted vs. the number of missing linkers (X-axis); in contaminated cavities, 1 additional molecule of terephthalic acid (TA) is added. (**b**) Inverse Laplace transform for the N-O rotational autocorrelation function of the TEMPO in defective cavities. The numbers of missing linkers are indicated; in addition, the black line shows the reference data for the ideal, non-defective cavity.

The obtained MD data show that the rotational correlation time of the radical increases when any of the above-mentioned modifications (I–II) are introduced into the UiO-66 cavities. As expected, although the removal of linkers increases the free volume available for TEMPO, the interaction with OH/OH_2_ groups has a more significant impact on the rotational correlation time, leading to a deceleration of the radical’s rotation (Figure 3a). It is also worth noting that the autocorrelation function for the radical’s motion in modified cavities is poorly described by a single decaying exponential. This suggests that the radical undergoes multiple types of motion at different time scales (Figure 3b).

Figure 3 clearly shows that the presence of defects leads to a deceleration of TEMPO rotation, and we observed a progressive increase in the rotational correlation time vs. the number of missing linkers. Furthermore, the incorporation of TA molecules instead of missing linkers in the same cavity additionally slows down and nearly completely immobilizes the radical. The inverse Laplace transform (Figure 3b) shows a probability distribution of rotational correlation times and features a single peak for the ideal cavity and a broader distribution when some linkers are removed (no TA was added for data shown in Figure 3b). In the case of two missing linkers, two distinct peaks can be observed: one at short times (τ_c_ ~ 0.1 ns) and another one at longer times (τ_c_ ~ 1.0 ns), corresponding to librations at small angles and jumps between MOF windows. As the third linker is removed, the peak with a longer correlation time shifts to the right. In general, the trend shown in Figure 3b corresponds to a progressively more efficient slow-down of the radical upon consecutive removal of linkers in defective cavities. Note that for contaminated cavities, a similar construction of Laplace-transform distributions was not performed due to the insufficient lengths of the MD trajectories.

In order to compare the obtained MD modeling results with the EPR-derived rotational correlation times, we list them in Table 1 and Table 2 (the latter is taken from the previous work [32]), respectively. Note that direct comparison of the absolute τ_c_ values obtained by MD and EPR might be ambiguous due to different methodologies employed to extract this parameter. Because of this, we have conducted an additional benchmarking experiment (see Supplementary Materials), which allowed us to conclude that maximum deviations between τ_c_ obtained by MD and EPR do not exceed a factor of three and, importantly, MD tends to overestimate the τ_c_ value.

A comparison of EPR and MD data indicates that initially, before applying mechanical stress, TEMPO radical is likely localized in defective cavities of UiO-66. This conclusion follows from a direct comparison of MD- and EPR-derived τ_c_ values and is further strengthened by a tendency of MD to overestimate τ_c_ (see Supplementary Materials). Indeed, the experimental τ_c_ = 1 ns for TEMPO@UiO-66 before pressurization (Table 2, first line) is much higher than the τ_c_ = 0.2 ns calculated for an ideal cavity, which may be an overestimated value (Table 1, first line). The MD-calculated correlation time obtained for the cavities with two missing linkers provides the best correspondence to the experimentally obtained value. Therefore, we suggest that in as-synthesized TEMPO@UiO-66, most probably, the radical is localized in the cavity with one or two missing linkers. The average number of missing linkers in TEMPO@UiO-66 particles measured by TGA was 2.5 per cavity. This can be rationalized by the overall large probability of such cavities in UiO-66 and by possible sterical factors and interactions operating during the inclusion of TEMPO into self-assembling MOF.

The trends demonstrated in Figure 3 provide several possible explanations for the exceptional pressure sensitivity of TEMPO@UiO-66 detected by EPR. First of all, Figure 3a shows that, in both defective and contaminated cavities, an increased amount of the missing linkers leads to deceleration of radical rotation (increase in τ_c_ value). While τ_c_ serves as a phenomenological parameter characterizing molecular rotational diffusion, we notice that defects and contaminants can significantly influence its value through several mechanisms. Contaminants decrease the free volume for the rotation of radicals and restrict their mobility, naturally leading to a decrease in rotational velocity and an increase in the rotational correlation time. The effect of missing linkers is less clear than that of contaminants; on the one hand, they increase the free volume and should increase the rotation speed, but on the other hand, they may lead to the onset of specific interactions with the missing linkers sites and corresponding deceleration of rotation. Thus, as follows from Figure 3a, contaminated cavities (with TA molecules) have much larger τ_c_ compared to just defective cavities. The values of τ_c_ higher than 10 ns correspond to immobilized EPR spectra; therefore, the generation of missing linker defect along with the corresponding contamination of the cavity explains the onset of immobile fraction in EPR spectra for the cavities having 1–3 missing linkers (Figure 3a, black). Even without contaminants, the trend in Figure 3a (red) shows that the radical should slow down its rotation when more missing linker defects are introduced. Thus, we conclude that the application of pressure leads to a generation of cavities with more than two missing linkers, to contamination of already existing defective cavities by MOF decomposition products, most likely being fully or partly unbound TA molecules, or by the interplay of these two processes. This constitutes the putative mechanism of high pressure sensitivity of TEMPO@UiO-66 materials with EPR detection.

The above-mentioned trends also allow us to explain all EPR observations on TEMPO@UiO-66 subjected to external pressure. Two model configurations of the UiO-66 cavities—defective cavities and contaminated cavities formed upon mechanical stress—can explain the coexistence of the two radical fractions observed in the EPR experiments. The mobile radical fraction shows a slight, gradual deceleration of movement as pressure increases. This is coherent with MD simulations indicating that the mobile fraction decelerates slightly as the number of missing linkers per cavity increases. Thus, the application of mechanical pressure leads to an increase in the number of missing linkers in defective cavities, supporting the idea that MOF degradation under mechanical stress begins in these defective cavities. The immobile fraction, which grows progressively with the value of applied pressure, corresponds to the TEMPO radicals localized in contaminated cavities, whose number naturally increases upon pressure-induced decomposition of MOF.

Finally, we notice that MD calculations in defective cavities (Figure 3b) confirm that the mobility of the ensemble of radicals becomes not uniform when more missing linkers are introduced. Namely, MD data show a distribution of τ_c_ values that broadens and shifts to the higher τ_c_ (i.e., slower rotation) when more missing linkers are introduced. This might alone explain the onset of two fractions observed in EPR; however, the absolute values of the rotational correlation times are in favor of the formation of contaminated cavities.

**Figure 4:**
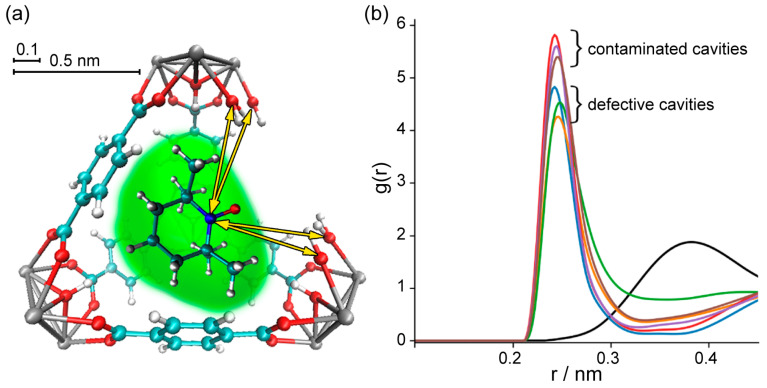
Radial distribution function (RDF) calculated for N atoms of TEMPO and OH/OH_2_ groups of Zr cluster at defect site. (**a**) Sketch of TEMPO@UiO-66 explaining measured distances. (**b**) RDF: groups of colored plots that correspond to TEMPO in defective UiO-66 cavity with different amounts of missing linkers/added TA molecules. The black curve corresponds to ideal UiO-66 cavity; in this case, RDF was calculated for N atom of TEMPO and OH groups of Zr cluster.

To investigate TEMPO mobility in different types of cavities in greater detail, we performed an analysis of the radial distribution function (RDF) for N atoms of TEMPO and OH/OH_2_ groups of the Zr cluster (Figure 4). The RDF, also denoted as g(r), describes the probability of finding a nitrogen atom of TEMPO at a distance r from the hydroxyl groups of UiO-66. For the analysis of RDF, we selected the nitrogen atom of TEMPO radical because it belongs to the NO group, which is the most polar moiety in the molecule. This group has a higher probability of forming specific interactions with various functional groups in the MOF structure. At the same time, we selected the hydroxyl groups of UiO-66 because these sites are commonly present at defect locations and are the most favorable for strong interactions with nitroxide.

The radial distribution function (Figure 4b) features a relatively sharp peak at ~2.5 Å for all defective structures, implying the presence of specific sorption sites; however, the defect-free UiO-66 (black line) does not feature this short-distance peak. Thus, in the case of defect-free UiO-66, we observed distant localization of the TEMPO NO-group relative to the Zr cluster. However, the introduction of missing-linker defects leads to a preferential location of the radical NO groups near Zr clusters. Upon adding one or more TA molecules to the cavity with missing linkers, we observed similar radical behavior, i.e., that the NO group noticeably approaches specific sites at the Zr clusters.

**Figure 5:**
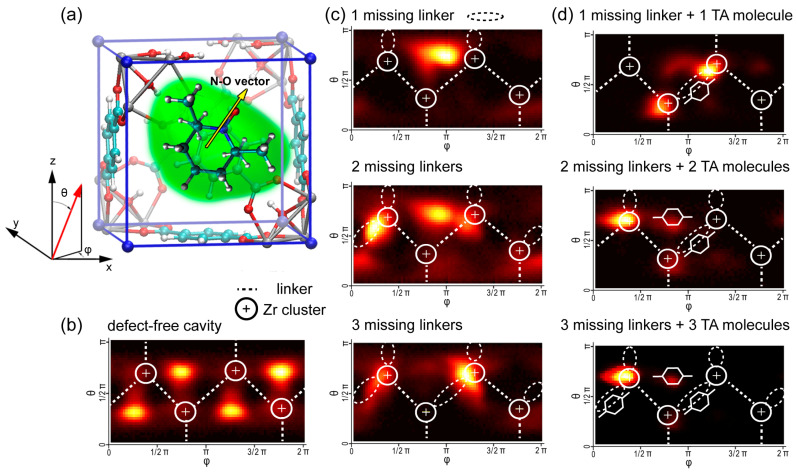
Orientation distribution of the N-O fragment of TEMPO radical: (**a**) Sketch of TEMPO@UiO-66 explaining angles orientation. (**b**) TEMPO inside defect-free UiO-66 cavity. (**c**) TEMPO inside defective UiO-66 cavity with different amounts of missing linkers. (**d**) TEMPO inside contaminated UiO-66 cavity with different amounts of missing linkers and with TA molecules added. Cone correction is applied (weighting with 1/sin θ).

To analyze the spatial distribution of the TEMPO radical inside UiO-66 cavities in even more detail, we examined the orientation distribution of the NO group (Figure 5). The probability density of specific orientations was visualized using two-dimensional heat maps, where the highest probability regions are represented by the most intense orange color.

The probability density analysis of molecular orientations in Figure 5 revealed that in defect-free UiO-66, TEMPO radicals predominantly occupy positions with their NO groups directed toward the cavity windows. Introducing missing-linker defects shifts this preference, and then the NO group tends to orient toward sites near the Zr clusters, at the same time maintaining a certain degree of stochastic reorientation within the cavity. When TA molecules were introduced into these defective cavities, we observed a strong preference for NO groups to become directed toward specific coordination sites on the Zr clusters. Thus, a combined analysis of the radial distribution function, orientation density, and correlation times explains the observed decrease in radical mobility through specific interactions with the binding sites in defective and/or contaminated MOF cavities.

## 3. Methods

Computational details: All MD simulations were carried out using the GROMACS package (version 2024.1; https://doi.org/10.5281/zenodo.15387018). For the model geometry, we chose 4 × 4 × 4 MOF cells with one embedded TEMPO nitroxide radical (Figure 1c). Flexible, AMBER-based force fields were used to describe the UIO-66 framework [47], and an ab initio-parameterized extension of the AMBER force field was employed for the study of large nitroxides, specifically for TEMPO [48,49].

MD simulations were performed in a triclinic periodic box with a side length of approximately 4.23 nm at room temperature. The initial geometries were generated using self-developed Python (version 3.12.2) scripts and the MDTraj library [50]. For each model geometry, a 100 ns NVT production run was performed at room temperature with a 1 fs time step. Prior to this, the system was relaxed, and pressure was equilibrated to 1 bar for 10 ns. A CSVR thermostat and Berendsen barostat were used, along with 4th-order PME for electrostatics. Coulombic and Van der Waals interactions were calculated with a 1.4 nm cutoff. Trajectory analysis was conducted using the MDTraj library [50], VMD [51], and Avogadro (version 1.98.1) [52,53]. Inverse Laplace transforms were performed using the NumPy Python library (version NumPy 1.26.0) [54].

The EPR-derived rotational correlation times (τ_c_) of TEMPO were taken from Ref. [32], where they were obtained by rigorous simulation of the CW EPR spectra using numerical solution of the stochastic Liouville equation (SLE) (see more details in Ref. [32]). This approach is incorporated into the EasySpin toolbox (version v5.2.36) for MATLAB (version 16.3.3) and accounts for both the rotational diffusion dynamics and magnetic interactions of the spin probe.

## 4. Conclusions

In this study, we investigated the exceptional pressure sensitivity of the UiO-66 framework with encapsulated nitroxide radical (TEMPO@UiO-66) using classical MD modeling with AMBER-based force fields. The high sensitivity of TEMPO@UiO-66 with EPR detection has been demonstrated by us previously, where first signs of the structural collapse were detected already at 0.06 GPa [32]; this observation has not been understood up to date. Herewith, we have provided a rationale for such high sensitivity, where the key factor is a localization of TEMPO radical in the cavities with missing linker defects. Such an approach allowed us to obtain theoretical rotational correlation times that are close to the EPR-derived ones for as-synthesized TEMPO@UiO-66. The onset of two fractions detected by EPR upon pressure application was then rationalized by an increase in the missing linkers amount and by contamination of cavities with the MOF decomposition products, most likely being fully or partly unbound terephthalic acid molecules.

The results of this work not only explain the higher EPR-detected sensitivity of TEMPO@UiO-66 to external pressure compared to the sensitivity of XRD but also provide useful tools for the future development and investigation of other MOF-based pressure sensors. For instance, variation in the missing linkers number, as well as the introduction of the contaminant molecules upon pressure-induced degradation, constitute a promising MD-based approach, as it is capable not only of explaining the experimental results but also allows the simulation of various new prospective MOF structures and optimization of a number of structural defects. Therefore, the results of this work can find future applications in the general design of MOF-based pressure sensors and mapping pressure distributions in polymers and other solids.

## Figures and Tables

**Table 1:** Rotational correlation times obtained by MD simulations of TEMPO@UiO-66 vs. a number of missing linkers in the cavity. For defective cavities, only the corresponding number of linkers was removed relative to the ideal structure, while for contaminated cavities, each missing linker was additionally compensated by a terephthalic acid (TA) molecule. For 0 missing linkers, the results for defective and contaminated cavities are therefore identical.

Number of Missing Linkers	Defective Cavity	Contaminated Cavity
	τ_c_/ns	τ_c_/ns
0	0.2	0.2
1	0.6	~10
2	1.2	~27
3	1.7	~41

**Table 2:** Rotational correlation times and corresponding fractions obtained by a simulation of X-band CW EPR spectra for TEMPO@UiO-66 [32]. Two fractions (mobile and immobile) contributed to the experimental spectra with the weights indicated in the ‘fraction’ columns. EPR studies were performed using TEMPO@UiO-66 samples containing an average of 2.5 missing linkers per cavity, as quantified by TGA [32].

Sample	Mobile Fraction		Immobile Fraction	
	τ_c_/ns	Fraction	τ_c_/ns	Fraction
Initial, 0 GPa	1.0	100%	-	0%
After, 0.06 GPa	2.2	90%	>100	10%
After, 0.13 GPa	3.2	49%	>100	51%

## Data Availability

The original contributions presented in this study are included in the article/Supplementary Material. Further inquiries can be directed to the corresponding author.

## Supplementary Materials

The following supporting information can be downloaded at https://www.mdpi.com/article/10.3390/molecules30102247/s1, Figure S1: Exponential decay of the autocorrelation function for the N–O bond vector of TEMPO molecules inside a ZIF-8 cavity loaded with paraxylene, Figure S2: X-band room-temperature CW EPR spectra of TEMPO@UiO-66 after exposure to varying pressures (0–0.13 GPa), Figure S3: Simulation of X-band room-temperature CW EPR spectrum of TEMPO@UiO-66 after exposure to 0.13 GPa. Comparison of Correlation Times Obtained from EPR and MD. References [19,49,55,56,57,58,59] are cited in the Supplementary Materials.
