# Supplementary material for: Pressure Sensitivity of UiO-66 Framework with Encapsulated Spin Probe: A Molecular Dynamics Study
Source: Molecules. 2025 May 21;30(10):2247. doi: 10.3390/molecules30102247 (PMC12113979; doi:10.3390/molecules30102247)
Supplement: Supplementary file 1 [file molecules-30-02247-s001.zip › molecules-3646140-supplementary.pdf]

SUPPORTING INFORMATION

Pressure Sensitivity of UiO-66 Framework with Encapsulated Spin Probe: A Molecular Dynamics Study

Dmitry V. Alimov [1,2], Artem S. Poryvaev [1] and Matvey V. Fedin [1,2,*]

1 International Tomography Center SB RAS, 630090 Novosibirsk, Russia; d.alimov@tomo.nsc.ru (D.V.A.); poryvaev@tomo.nsc.ru (A.S.P.)
2 Physics Department, Novosibirsk State University, 630090 Novosibirsk, Russia
* Correspondence: mfedin@tomo.nsc.ru;

Table of Contents

I. Comparison of Correlation Times Obtained from EPR and MD………………………………….. S2

II. EPR measurments……………………………………………………………………………….. S4

III. References………………………………………………………………………………….. S5

I. Comparison of correlation times obtained from EPR and MD

The comparison of the absolute values of the rotational correlation times (τ_c) obtained in EPR and MD requires justification. In fact, in both cases τ_c are phenomenological parameters obtained in certain approximation. In EPR spectroscopy this τ_c value is obtained using numerical simulation of CW EPR spectrum of nitroxide, employing Stocks-Einstein-Debye formula. In MD calculations τ_c is obtained from the exponential fitting of the autocorrelation function decay. Therefore, although all trends should be uniformly reproduced by both methods, the absolute values of τ_c might be somewhat different.

To evaluate possible scale of deviations between τ_c values obtained from EPR and MD, we considered a model system. Since UiO-66 is the MOF known for pronounced number of defects, it cannot be used for benchmarking. Another MOF, ZIF-8, is, other way around, known to be virtually free of defects, thus representing a proper model system to correlate τ_c values in EPR and MD.

Thus, we studied the TEMPO@ZIF-8 system, obtained following previously published procedures.[1] The rotational correlation time for TEMPO@ZIF-8 was calculated using exactly the same approach as that employed for TEMPO@UiO-66 in the present work (see description in the main text). Specifically, we determined the rotational correlation time by fitting the autocorrelation function of the radical fragment's vector (aligned along the N–O bond) to a decaying exponential.

For bare MOF (without guest solvent molecules) TEMPO@ZIF-8, our results for τ_c obtained using MD simulations agree well with experimental EPR-derived ones (~20 ps).[1] However, for bare TEMPO@ZIF-8 the τ_c value obtained from EPR is rather short, being on the detection limit. Therefore, to slow down radical rotation and obtain more reliable conclusions, we introduced 3 to 4 paraxylene molecules into each cavity. Such loading level was chosen, because 3–3.75 paraxylene molecules typically occupy each ZIF-8 cavity at room temperature.[2]

Molecular dynamics simulations were performed for the TEMPO@ZIF-8 + paraxylene as follows. The simulation parameters and TEMPO parameterization were consistent with those used in the main article. The ZIF-8 parameterization was adopted from Anikeenko et al.[3], while the paraxylene parameterization was generated using the AMBER force field via LigParGen.[4] The initial system geometries were constructed using PackMol.[5] For each configuration, 100 ns of MD trajectories were calculated, starting with 3 to 4 paraxylene molecules located within the TEMPO-containing pore of ZIF-8. Due to diffusion, the number of paraxylene molecules in the pore varied over time.

To ensure consistent analysis, trajectories were segmented into 5 ns intervals, and only segments where the number of paraxylene molecules within the pore remained constant were considered. The method used to count paraxylene molecules in the pore was similar to that described by Anikeenko et al.[3] Rotational correlation times were determined using the rotational autocorrelation function (ROTACF), averaged over all trajectory segments corresponding to each fixed paraxylene amount. The resulting ROTACF curves were fitted with a decaying exponential function. Fitting was performed using the gradient descent method by minimizing the standard deviation (see example in Figure S1). Since ROTACF values below 0.1 are less reliable due to noise, we used a cutoff of ROTACF > 0.1 and computed the standard deviation only in that region.

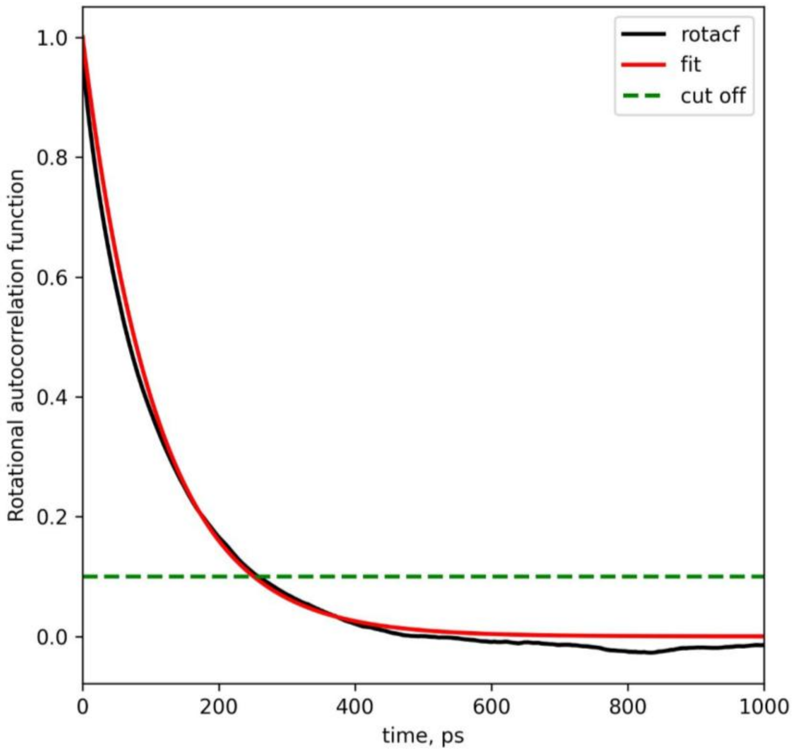

Figure S1. Exponential decay (**fit** – red line) of the autocorrelation function for the N–O bond vector (**rotacf** – black line) of TEMPO molecules inside a ZIF-8 cavity loaded with paraxylene (3 molecules per cavity). To improve the accuracy of our analysis, we applied a **cut-off** and only considered the first ~240 ps of data.

MD simulations yielded rotational correlation times of 109 ps and 350 ps for systems containing 3 and 4 paraxylene molecules per ZIF-8 cavity, respectively. These results are in good agreement with previously published EPR data, which reported a correlation time of 130 ps for TEMPO@ZIF-8 loaded with 3.3±0.3 paraxylene molecules.[2,6] Thus, τ_c value calculated using MD falls within ~85-270% range relative to the EPR-derived τ_c value. This means that, in conditions relevant to the present study, MD tends to overestimate τ_c value relative to the EPR-derived τ_c, but hardly more than by a factor of 3.

Note that this tendency to overestimate τ_c value in MD calculations only strengthens the conclusions for TEMPO@UiO-66 discussed in the main text. Indeed, MD calculations predict τ_c = 0.2 ns for ideal cavity, τ_c = 0.6 ns for a cavity with 1 missing linker, and τ_c = 1.2 ns for a cavity with 2 missing linkers. EPR data deliver τ_c = 1 ns for as-synthesized TEMPO@UiO-66. Since MD is prone to overestimate τ_c, it is undoubted that TEMPO must be localized not in ideal cavity, but in defective cavity with 1, 2 or more missing linkers.

II. EPR measurements

EPR measurements were performed using commercial Bruker EMX X-band EPR spectrometer at the Center of Collective Use "Mass spectrometric investigations" SB RAS. In all cases, powder solid samples were placed into quartz sample tubes (OD = 3.8 mm). Continuous wave (CW) EPR spectra were recorded at conditions avoiding unwanted modulation broadening and microwave saturation. For all simulations, EasySpin toolbox for Matlab was used[7]. To apply controlled external pressure to TEMPO@UiO-66 powders, we used a Specac hydraulic pellet press equipped with a pressure gauge. The powder sample was placed between pellet dies (13 mm diameter), and pressure was gradually increased over 2–3 minutes, then held stable for an additional 10 minutes before measurement. Room-temperature EPR measurements (298 K) were conducted on both the initial TEMPO@UiO-66 sample and samples subjected to different mechanical pressures (see Figure S2).

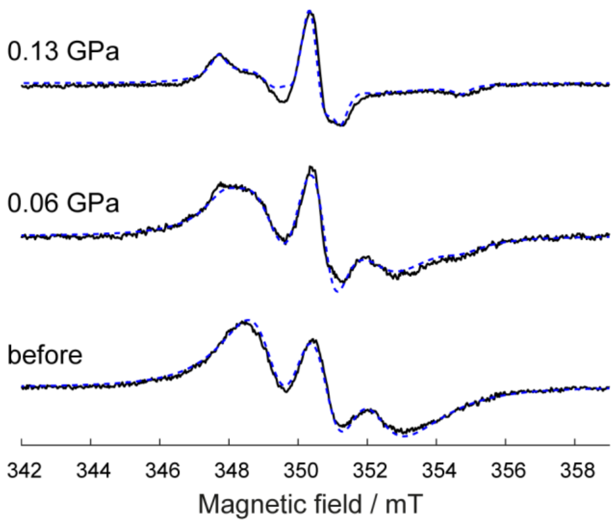

Figure S2. X-band room-temperature CW EPR spectra of TEMPO@UiO-66 after exposure to varying pressures (0–0.13 GPa). Blue dotted lines in (a) show simulated EPR spectra using the following spectroscopic parameters: g-tensor mobile fraction g = [2.0090 2.0085 2.0055] and immobile fraction g = [2.0090 2.0080 2.0040], hyperfine interaction tensor mobile fraction A = [0.52 0.52 3.80] mT and immobile fraction A = [0.62 0.62 3.50], correlation time and contribution of mobile and immobile fraction are shown in the main text.

The mobile and immobile fractions of nitroxide were determined by spectral superimposition of these components (Figure S3).

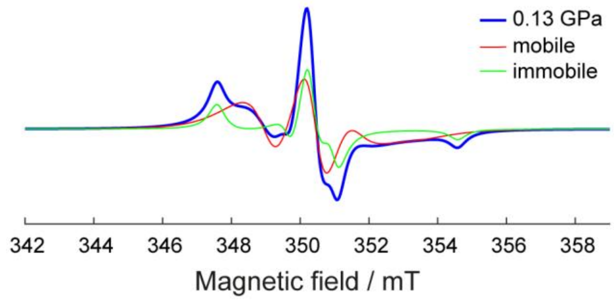

Figure S3. Blue line shows simulation of X-band room-temperature CW EPR spectrum of TEMPO@UiO-66 after exposure to 0.13 GPa. Red line in show simulated EPR spectrum using spectroscopic parameters for mobile fraction g = [2.0090 2.0085 2.0055], A = [0.52 0.52 3.80] and τ_c = 3.2 ns, Green line in show simulated EPR spectrum using spectroscopic parameters for immobile fraction g = [2.0090 2.0080 2.0040], A = [0.62 0.62 3.50] and τ_c > 100 ns.

III. References

[1] D. M. Polyukhov, A. S. Poryvaev, S. A. Gromilov, M. V. Fedin, Precise Measurement and Controlled Tuning of Effective Window Sizes in ZIF-8 Framework for Efficient Separation of Xylenes. *Nano Lett.* **2019**, *19*, 6506-6510.

[2] D. M. Polyukhov, A. S. Poryvaev, A. S. Sukhikh, S. A. Gromilov, M. V. Fedin, Fine-Tuning Window Apertures in ZIF-8/67 Frameworks by Metal Ions and Temperature for High-Efficiency Molecular Sieving of Xylenes. *ACS Appl. Mater. Interfaces* **2021**, *13*, 40830–40836.

[3] A. M. Sheveleva, A. V. Anikeenko, A. S. Poryvaev, D. L. Kuzmina, I. K. Shundrina, D. I. Kolokolov, A. G. Stepanov, M. V. Fedin, Probing Gas Adsorption in Metal–Organic Framework ZIF-8 by EPR of Embedded Nitroxides. *J. Phys. Chem. C* **2017**, *121*, 19880-19886.

[4] https://traken.chem.yale.edu/ligpargen/

[5] https://m3g.github.io/packmol/

[6] A. A. Efremov, A. S. Poryvaev, D. M. Polyukhov, R. Z. Sagdeev, M. V. Fedin, Anisotropic Rotation of TEMPO Radical in the Cavities of Metal–Organic Framework ZIF-8 Induced by Guest Solvents. *Appl. Magn. Reson.* **2023**, *54*, 93–105.

[7] S. Stoll, A.Schweiger, EasySpin, a comprehensive software package for spectral simulation and analysis in EPR. J. Magn. Reson. 2066, 178, 1, 42-55
